# Concentration of the genera Aspergillus, Eurotium and Penicillium in 63-μm house dust fraction as a method to predict hidden moisture damage in homes

**DOI:** 10.1186/1471-2458-9-247

**Published:** 2009-07-17

**Authors:** Christoph Baudisch, Ojan Assadian, Axel Kramer

**Affiliations:** 1 State Health and Social Office of Mecklenburg-Pomerania, Branch Office Schwerin, Germany; 2 Clinical Institute for Hygiene and Medical Microbiology of the Medical University of Vienna, Department of Hospital Hygiene, Vienna General Hospital, Austria; 3 Institute for Hygiene and Environmental Medicine, Ernst Moritz Arndt University, Greifswald, Germany

## Abstract

**Background:**

Quantitative measurements of mould enrichment of indoor air or house dust might be suitable surrogates to evaluate present but hidden moisture damage. Our intent was to develop a house-dust monitoring method to detect hidden moisture damage excluding the influence of outdoor air, accumulated old dust, and dust swirled up from room surfaces.

**Methods:**

Based on standardized measurement of mould spores in the 63-μm fraction of house dust yielded by carpets, the background concentrations were determined and compared to simultaneously obtained colony numbers and total spore numbers of the indoor air in 80 non-mouldy living areas during summer and winter periods. Additionally, sampling with a vacuum-cleaner or manual sieve was compared to sampling with a filter holder or sieving machine, and the evaluative power of an established two-step assessment model (lower and upper limits) was compared to that of a one-step model (one limit) in order to derive concentration limits for mould load in house dust.

**Results:**

Comparison with existing evaluation procedures proved the developed method to be the most reliable means of evaluating hidden moisture damage, yielding the lowest false-positive results (specificity 98.7%). Background measurements and measurements in 14 mouldy rooms show that even by evaluating just the indicator genera in summer and winter, a relatively certain assessment of mould infestation is possible.

**Conclusion:**

A one-step evaluation is finally possible for house dust. The house-dust evaluation method is based on analysis of the indicator genera *Aspergillus*, *Eurotium *and *Penicillium spp*., which depend on the total fungal count. Inclusion of further moisture indicators currently appears questionable, because of outdoor air influence and the paucity of measurements.

## Background

Moisture damage in homes can lead to proliferation of ubiquitous moulds [1]. The healthy consequences are disturbances to well-being, such as bad odors or headaches, and even chronic illnesses like asthma and allergies [2-5]. While the moisture level in a room expressed as relative humidity can easily be measured with a hygrometer, this method is often not suitable for detecting the presence of localized, hidden moisture. Measuring the mould load in air or house dust can be used as an indirect indicator for present but hidden moisture damage. However, because mould spores simultaneously disperse via airborne dust and settle in the environment, results of airborne dust measurements can vary for several factors, most importantly the quantity of settled dust – that is, already accumulated old dust (of indefinite age) and newly settled dust (of definite age) [6] – and how strong dust turbulence is in a particular environment. The degree of cleanliness, the frequency of a room being used, the inflow of outdoor air, and the air circulation all exert an influence on indoor-air dust measurements. Hitherto, these aspects have received little attention.

We modified the method for processing moulds previously presented by Butte et al. [7] by additionally sieving house dust for moulds in order to reduce the sampling error which might occur by possible dilution with sand. Briefly, in a pilot study, we substituted the conventional dust analysis method for mould load in total dust [8] with an analysis of the sieved 63-μm dust fraction and assessment of fungal indicator genera [9], in order to reduce measurement uncertainty [Baudisch C, Kramer A, Assadian O: Evaluation of errors and limits of the 63-μm house-dust-fraction method, a surrogate to predict hidden moisture damage. submitted] particularly in cases of questionable loads [10-13]. Other authors have also examined carpet-dust samples for moulds [5,14-23], with sieve sizes differing from 2500 to 250 μm [24-30].

In 2001, the German Federal Environmental Office ("Umweltbundesamt") initiated a research project, the UFOPLAN study, to determine the background concentrations of indoor mould in order to standardize mould measurement procedures in non-mouldy homes [31-34]. This included the examination of the 63-μm fraction of house dust. Gabrio et al [31] modified the sampling method by using a filter holder and a sieving machine instead of a vacuum-cleaner and manual sieving to reduce the effort involved in obtaining house-dust samples (cutting open the vacuum bag in the laboratory while wearing a face mask and protective suit; manual sieving) and to possibly improve the reproducibility.

The evaluative power of a two-step versus a one-step assessment model for house dust was determined. In the one-step assessment model, one concentration limit, below the background levels and above the damage levels, are set. The two-step assessment model employs a lower limit (for house dust corresponding to the 95th percentile) and an upper limit (for house dust corresponding to triple the 95th percentile). Below the lower limit, only background levels are expected, and above the upper limit only damage levels. Between the lower and upper limits lays a range of questionable loads.

By obtaining information on the specificity (proportion of true negative findings) of the mould measurements taken from house dust and indoor air [32,34], we intended to identify the most accurate method for assessing the mould load in houses. The question was also whether it is possible to evaluate moisture damage by indicator genera, since Flannigan et al. [35] and Flannigan and Miller [36] saw the genera *Aspergillus *and *Penicillium *as typical indoor moulds. Würtz et al. [37] and Petola et al. [38] also found an association of these indicator genera with moisture damage.

Based on these results [9,32], the aim of the present study was to develop a house-dust assessment method to detect the presence of already existing hidden moisture damage, while largely excluding the influence of outdoor air, old dust, and dust swirled up from the room surfaces.

## Methods

In accordance with the UFOPLAN study design [31-34], we specifically wanted to employ the filter-holder method for the 63-μm fraction of house dust in 80 non-mouldy household living rooms to determine and compare the mould levels in winter (2002/2003) and summer (2003), and derive the background concentration from the sum-frequency distributions of the measured values. Using our preliminary results and the one-step method, sampling with a vacuum-cleaner or manual sieving was compared to sampling with the filter holder or sieving machine

After one week without vacuuming, samples were collected. Rooms were defined as non-colonized if no moisture damage was visible and no plants or caged animals were present. The living areas had carpeted floors. Generally, a carpeted floor accumulates fungal spores better than a smooth, hard floor. Additionally, more dust can be obtained.

To collect dust, the filter holder [6] with a polycarbonate filter (diameter 5 cm) was used. Samples were collected within 10 minutes from a 2 m^2 ^sized sampling area, whereby one single sample combined all 4 quadrants of the total sampling area, each 0.5 m^2 ^in size. Sampling-nozzle openings of 6 and 10 mm were used. In order to maintain a constant suction speed, flow rates of 15 l/min and 42 l/min were set. Subsequent sieving was performed using a machine (1.50 mm amplitude at 50 Hz, corresponding to 7.6 g sieve-acceleration; 1 g = 9.81 m/s^2^) possessing a special set of sieves with 3 serial sieve sizes (400, 150 and 63 μm, diameter 5 cm), agate balls of different diameters to support sieving, and a flow rate of 1 l/min. To determine the number of colony forming units (cfu) of moulds, the 63-μm house-dust sample was suspended with 100 times its weight (e.g., 100 mg in 10 ml) in 0.89% NaCl solution to which 0.001% Tween 80 was added. After shaking for 30 minutes with a round shaker at 500 rpm, 0.1 mL was inoculated onto each of three DG18 agar plates (Heipha GmbH, Eppelheim, Germany), either undiluted or as 1:10 and 1:100 dilutions. The same amount and dilutions were inoculated onto 3 malt-extract agar plates (Heipha GmbH, Eppelheim, Germany) at 25 ± 3°C and 37 ± 1°C to detect further moisture indicators (e.g., Acremonium spp, Stachybotrys spp or Chaetomium spp) [9,31,32]. Inoculation and incubation of samples was performed a few days after sampling due to shipment by post.

House-dust samples were evaluated for the indicator genera *Aspergillus spp*., *Eurotium spp*. or *Penicillium spp*. The perfect stages of *Aspergillus spp*., *Eurotium spp*. (class: Ascomycota) with sexual reproduction, and *Aspergillus spp*. with vegetative reproduction (class: Deuteromycota) were taken together as one group. Evaluation was based on the dilution level, whose optimal plate colonization lies between 20 and 40 cfu/plate or 10 and 100 cfu/plate [39]. Counts were made between days 2 and 10 of incubation. The average values for each species were taken from the 3 plates of the optimum dilution level. This yielded a summer and a winter dust sample for each living room examined. To identify the indicator genera, preferential the results from the DG-18 -agar were used. The basis for differentiating the moulds consisted of comparing to reference strain collections and the available literature [e.g. [40-44]].

Before the house-dust measurements with the filter holder were performed, the mould concentration in indoor and outdoor air was determined with the air-sampling system MAS 100 (Merck Darmstadt Germany; impaction method; aspiration unit with perforated lid [400 holes] in sampling head), as was the number of mould particles (total spore count method using Holbach's impactor with slit sampling impactor; Umweltanalytik Holbach GmbH Wadern Germany) [34].

## Results

**Figure 1:**
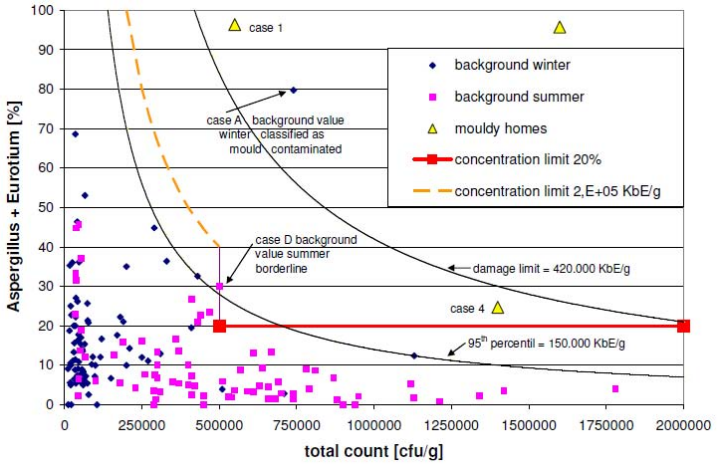
**Percent concentration of sum of Aspergillus and Eurotium spp. in reference to the total count**.

**Figure 2:**
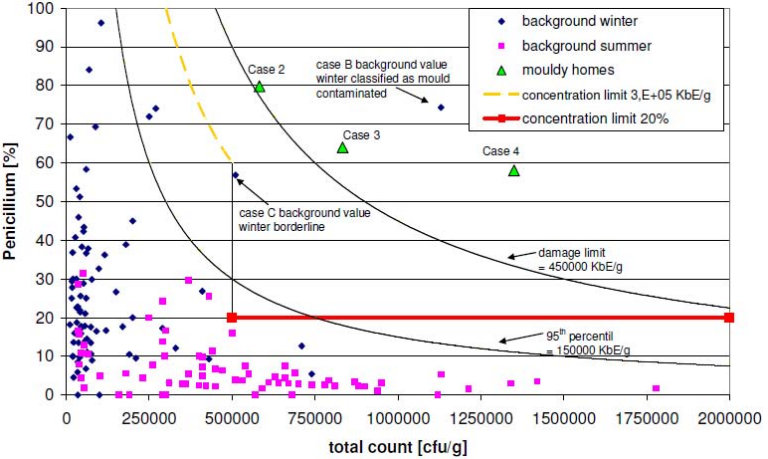
**Percent concentration of Penicillium spp. in reference to total count**.

Based on the pre-existing one-step assessment model [9] and the sampling results of the UFOPLAN study [32,34], a new one-step assessment model was developed for the evaluation of cultivable moulds in the 63-μm fraction of house dust (Table 1). Applying this model, moisture damage has a high probability of occurrence if at least one *concentration limit *for the indicator genera *Aspergillus spp*., *Eurotium spp*. (Figure 1), or *Penicillium spp*. (Figure. 2) is exceeded, depending on the total fungal counigut (number of cultivable mould spores and mycelium fragments; hereafter termed total count).

**Table 1:** Evaluation of mould in 63-μm house dust fraction; modified one-step assessment model

Corresponding total count without yeast (cfu/g)	Concentration limit, indicator genus	
	
	*Aspergillus + Eurotium spp.* (cfu/g) or proportion (%)	*Penicillium spp.* (cfu/g) or proportion (%)
0 – 500,000	≥ 200,000 cfu/g	≥ 300,000 cfu/g
500,000 – 2,000,000	≥ 20%	≥ 20%
> 2,000,000	≥ 420,000 cfu/g	≥ 450,000 cfu/g

Specificity (true negative result in non-mouldy homes = 100% minus proportion of false positive results in non-mouldy homes) can be used as a criterion for the quality of an assessment model. Applying the recommended one-step assessment model (Table 1) for the measurements from non-mouldy homes, 2 false positives resulted among winter samples (case no. A and B, Figure. 1 and 2) and 2 borderline values each in the winter and summer samples which could be assigned to the range of samples without fungal growth (case no. C and D, Figure. 1 and 2), resulting a specificity of 98.7%.

Table 2 presents analyzed cases (n = 14) of mouldy homes with mouldy walls (cases 2, 4, 6, 10, 12), moulds in hollow spaces (cases 3, 7, 8), wet carpets (cases 1, 13, 14), wet cellars (cases 9a, 9b) and highly mouldy or wet adjacent rooms (cases 5, 11). The corresponding count and percent proportion of the indicator genera *Aspergillus spp*., *Eurotium spp*., or *Penicillium spp*. and the total count are given. House-dust measurements from all mouldy homes exceeded the *concentration limits *for the indicator genera, and were markedly greater than the background levels. For the few actually mouldy homes monitored to date, the sensitivity (true positive result in a mouldy home = 100% minus proportion of false negative results in a mouldy home) of the house-dust method thus lies at 100%, although the mould colonization was often not visible (Table 2).

**Table 2:** Confirmation of the assessment model in mouldy homes (sampling between 1998 and 2004)

**Case No**	**General conditions, site inspection**	moulds total	*Aspergillus *+ *Eurotium *spp		*Penicillum *spp	
		cfu/g	**%**	**cfu/g**	**%**	**cfu/g**

1	No visible mould, flooded basement hallway had been dried (winter)	550,000	**96**	**530,000**	4	20,000

2	No visible mould, remove wall mould, carpet not renovated	580,000	9	53,000	**80**	**470,000**

3	No visible mould, ground floor, freshly lacquered wooden floorboards, earth underneath, cellulose blown in as insulation, musty odor, empty room, no carpet	830,000	6	50,000	**64**	**530,000**

4	Wall mould, outside wall mouldy	1,400,000	**25**	330,000	**58**	**780,000**

5	No visible mould, flooded archives in basement, basement office still dry, door always open	1,600,000	**96**	**1,500,000**	2	30,000

6	Wall paper moldy, stale/musty odor	2,300,000	**50**	**1,100,000**	12	280,000

7	No visible mould, children's room, plasterboard interior insulation mouldy	3,500,000	13	**470,000**	**37**	**1,300,000**

8	No visible mould, bedroom, mouldy under the flooring	3,600,000	12	**430,000**	**32**	**1,200,000**

9a	No visible mould, damp basement wall of a teens' club, filter-holder sampling, parallel measurement to case 9b, in both cases 2.6 million cfu *Wallemia spp*./g	3,800,000	**25**	**970,000**	0	20,000

9b	No visible mould, vacuum-cleaner sampling, parallel measurement to case 9a	3,900,000	**21**	**810,000**	2	70,000

10	Wall mould, 4 m^2^, upper floor vacuumed	5,000,000	**87**	**4,300,000**	7	350,000

11	No visible mould, flooding in basement library, renovation without replacing fitted carped, formerly dry area (summer)	7,500,000	15	**1,100,000**	5	400,000

12	Wall mould	8,300,000	19	**1,600,000**	**40**	**3,300,000**

13	Flooded basement room, now dry but not renovated	9,700,000	**85**	**8,300,000**	4	370,000

14	No visible mould, flooding in basement library, renovation without replacement of fitted carped, formerly flooded area (summer)	16,000,000	20	**3,100,000**	11	**1,700,000**

## Discussion

To evaluate the measured values from the UFOPLAN study [32], the 95^th ^percentile (step one) of background levels (n = 157) and 3 times the 95^th ^percentile ("damage limit", step two) of these are used, which were obtained from the sum-frequency distribution for cultivable fungi from carpet dust samples (<63 μm) (Trautmann et al.'s two-step assessment model for house dust [32]). The 95^th ^percentile and the damage limits are depicted in Figureures 1, 2, 3 and 4 for the selected indicator genera *Aspergillus spp*., *Eurotium spp*., or *Penicillium spp.*, along with the results of the UFOPLAN study (background winter/summer), the results of the load measurements (mouldy homes, Table 2), and the concentration limits of our one-step assessment model (Table 1). According to Trautmann et al. [32], mould concentrations in house dusts below the 95^th ^percentile represent background loads. Values in excess of damage limits are usually found only for mould-loaded (Table 2) samples. In the two-step assessment models (dust and air [32,34]), a questionable range exists between the lower and the upper limit.

**Figure 3:**
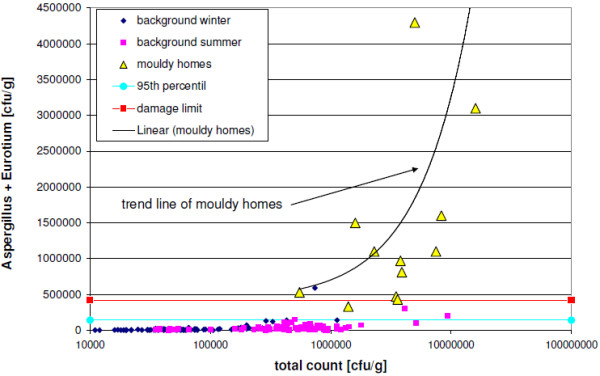
**Concentrations of sum of Aspergillus and Eurotium spp. against the total count in cfu/g; complete measurement range**.

**Figure 4:**
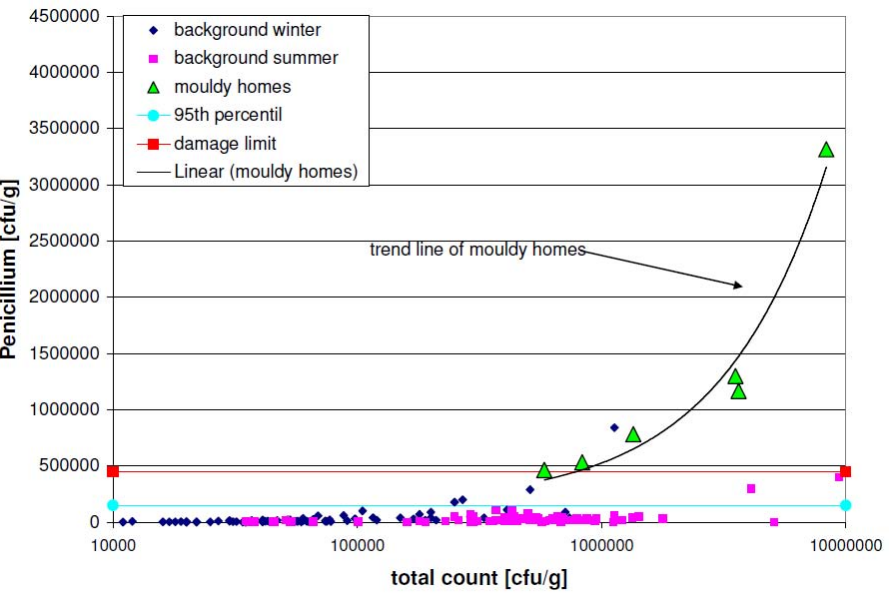
**Concentrations of Penicillium spp. against the total count in cfu/g; complete measurement range**.

Evaluating the background measurements for the indicator genera using the two-step assessment model [32], two false-positive values were found above damage limits, but 8 background values fell in the questionable range, and 2 were found to be borderline. In contrast, the two dust-evaluation models are identical for the indicator-genera evaluation above damage limits.

The specificity of the methods studied are as follows: 1. House-dust measurement (63 μm) by our one-step model with concentration limits for two indicator genera depending on total count: specificity 98.7%; 2. house-dust measurement (63 μm) by the two-step model, all moisture indicators: specificity 74%; 3. air monitoring, culturing procedure or total airborne spore measurement: specificity in the range of 80%. These results demonstrate the great advantage of the house-dust evaluation approach examined here.

In keeping with EN 14988 [8] for assessing mould levels in house dust, only the total count with 10-times greater concentration limits (due to the difference between total dust and the 63-μm dust samples up to factor 10) was initially used, but it excluded yeast. With high outdoor-air loads in summer, this approach led to limits being exceeded, although no moisture damage was present. The further development of the assessment model rests on two facts: first, the background load of all species increases in summer. This is compensated through a percent evaluation of moulds relative to the total number. Second, the indicator genera *Aspergillus spp*., *Eurotium spp*., or *Penicillium spp*. occur "relatively" constantly throughout the year in rooms [25]. Hence, the increased amounts of these genera indicate moisture damage. The percent evaluation of indicator genera is intended – above a base load in winter – to eliminate the summer influence [9]. It was not apparent from the available data up to that point that this also has an upper limit.

In the linear depiction of the indicator genera, the background levels generally lie under the 95^th ^percentile (Figures 3 and 4). In the 3 total-count areas (Table 1), however, quantitatively different results are found, which become more obvious in the depiction of percentages (Figures 1 and 2). Up to a total count of 500,000 cfu/g, higher levels/very high proportions (%) of the indicator genera occur especially in winter, with the genus *Penicillium spp*. contributing higher values. With three exceptions (n = 78), background values in winter all lie below the total count of 500,000 cfu/g. Mouldy homes have to date not been found in this range (Table 2). Thus, the original one-step assessment [9] is confirmed, which defined total counts of < 500,000 cfu/g as background loads. This method agrees with the assessment described by EN 14988 [8], which distinguishes between samples with intermediate and high fungal growth, with total counts of at least 50,000 cfu/g (levels in this study 10 times higher due to sieving). For the total-count range up to 500,000 cfu/g, the assessment by indicator genera is adjusted to the present results (moisture damage by exceeding concentration limits for *Aspergillus spp*. and *Eurotium spp*. > 200,000 cfu/g or *Penicillium spp*. > 300,000 cfu/g; see Figures 1 and 2 and Table 1).

At a total count of 500,000 cfu/g, a drop to lower values is evident (Figures 1 and 2), which is apparently seasonal. In the total-count range of 500,000 cfu/g to 2,000,000 cfu/g, the proportions of indicator genera [%] decrease with increasing total count or the levels [cfu/g] remain relatively constant below the 95^th ^percentile. These are primarily summer background values, which, although diluted by other outdoor airborne spores such as *Cladosporium spp*, were not dramatically influenced. Background levels are percentually differentiated from mouldy home levels (moisture damage if one *concentration limit *for the indicator genera ≥ 20% of the total count, Table 1, Figures 1 and 2). A more stringent assessment than that using the 95^th ^percentile results from this, up to a total count of ca. 700,000 cfu/g. In the next consecutive total-count range up to 2,000,000 cfu/g, the percentual assessment yields a transition to damage limits (Figures 1 and 2), which should avoid the possible massive influence of outdoor air. However, in the total-count range of 0 to 2,000,000 cfu/g, damage limits are meaningless for the indicator genera (see Figures 1 and 2), because their concentrations are still close to the 95^th ^percentile. Thus, the two-step assessment is inapplicable.

The trend lines in Figures 3 and 4 indicate that in mouldy homes, the concentration of indicator genera increases with increasing total count, and progresses increasingly away from damage limits. Thus, borderline mouldy homes can be expected in a total-count range of 500,000 to 700,000 cfu/g. The percentual assessment model is particularly sensitive for this total-count range.

In the third total-count range of > 2,000,000 cfu/g, only 3 summer values (Figures 3 and 4) were heavily influenced by outdoor air and lay below damage limits, which is used to delimit this range (assessment criteria: level for *Aspergillus spp*. and *Eurotium spp*. > 420,000 cfu/g or *Penicillium spp*. > 450,000 cfu/g, Figures 3 and 4, Table 1).

Using the method introduced here and the parameters examined, correlations between impact on health and total dust load – unlike in other epidemiological studies [45] – are not possible, because previous vacuuming eliminated the influence of old dust and cleaning status, and any sand influence was negated by sieving [Baudisch C, Kramer A, Assadian O: Evaluation of errors and limits of the 63-μm house-dust-fraction method, a surrogate to predict hidden moisture damage. submitted]. If the method is to be used in epidemiological studies, it would be advantageous to omit vacuuming before sampling, in order to include the given cleaning habits (old dust). Mould exposure in interior spaces is not only influenced by moisture damage, but also by old dust [45]. However, it is to be expected that the indicator genera – with their enormous reproductive proclivity under damp conditions and their ease of airborne dispersal – in water-damaged dwellings will be much easier to detect in air and house dust than the more slowly reproducing and less easily airborne hydrophilous mould species, such as Stachybotrys spp. or Chaetomium spp. [46].

Sieving to 63 μm will also improve the quantitative proof of other house-dust components. Toxins [47] or allergens are found in the finer fraction (< 63 μm) of house dust [48]. Through this enrichment by sieving, a stronger (and as a rule more certain) signal is obtained, and homogenizing eliminates the error influence of sand Baudisch C, Kramer A, Assadian O: Evaluation of errors and limits of the 63-μm house-dust-fraction method, a surrogate to predict hidden moisture damage. submitted].

## Conclusion

Compared to other measurement methods, the present results show that a one-step indicator genera assessment for the 63-μm fraction of house dust (based on total colony count from the measurement and assessment method by Baudisch et al. [9]) provides the best representation of the occurrence of mould in homes (highest specificity, 98.7% and highest sensitivity, 100%). Furthermore, it is possible to integrate seasonal influences – such as the strong influence of outdoor-air influx in summer and enrichment effects of the indicator genera in total colony counts of up to 500,000 cfu/g especially in winter – in the evaluation with regard to total colony counts. Thus, the present method does not depend on season.

In the authors' opinion, however, absolutely certain information on exposure to airborne dust (mould spores) in living areas is still impossible due to the undefined turbulence and method-dependency of results. Nevertheless, indoor airborne mould measurements provide the best means of determining the actual level to which the individual is exposed. In dealing with individual health problems (allergy, infection, intoxication), it is always also necessary to completely identify the fungal species, in addition to monitoring the air in living areas (e.g., direct method). For determining moisture damage alone, it is often sufficient to take material samples (e.g., wallpaper) with a description of the extent of the affected area and/or building construction (investigation of source). An essential part of every assessor's evaluation – in addition to mould measurements (material/fabric, dust, air, total spores) – is the site inspection.

## Competing interests

The authors declare that they have no competing interests.

## Authors' contributions

CB and AK conceived the study. CB and AK designed and coordinated the study, CB, OA, and AK analyzed the data and wrote the first draft of the manuscript. OA finalized and manuscript. All authors helped to draft the manuscript and read and approved it in its final form.

## Pre-publication history

The pre-publication history for this paper can be accessed here:


